# Cancer Stem Cells in Head and Neck Cancer

**DOI:** 10.3390/cancers3010415

**Published:** 2011-01-20

**Authors:** Doyel Mitra, Stephen P. Malkoski, Xiao-Jing Wang

**Affiliations:** 1 Department of Pathology, University of Colorado Denver, AMC 12800 E. 19^th^ Ave., Aurora CO, 80045, USA; E-Mail: doyel.mitra@ucdenver.edu; 2 Department of Medicine, Division of Pulmonary Sciences and Critical Care Medicine, University of Colorado Denver, Aurora CO, 80045, USA; E-Mail: stephen.malkoski@ucdenver.edu

**Keywords:** chemoresistance, radioresistance, transplantation, tumorigenicity

## Abstract

Head and neck cancer (HNC) is the sixth most common malignancy world-wide, however the survival rate has not improved for the past 20 years. In recent years, the cancer stem cell (CSC) hypothesis has gained ground in several malignancies and there is mounting evidence suggesting CSCs mediate tumor resistance to chemotherapy and radiation therapy. However, the CSC theory is also challenged at least in certain types of cancer. Here we review the progress of CSC studies in HNC, which suggest that HNC conforms to the CSC model. The identified CSC markers and their tumor initiation properties provide a framework for the development of novel therapeutic strategies for HNC.

## 1. Introduction

**Figure 1.**
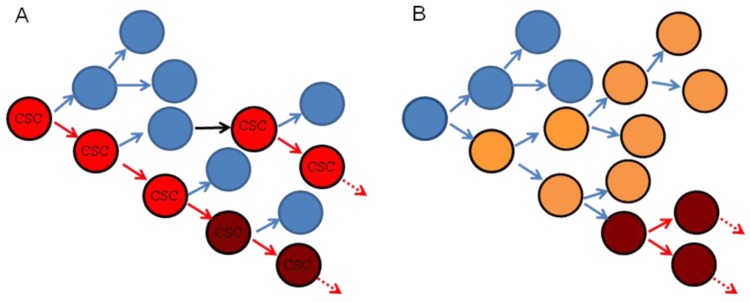
Cancer heterogeneity models. (**A**) Hierarchical model of Cancer Stem Cell (CSC). The CSC (in red) undergoes a division to give rise to a differentiated daughter cell (blue) and another CSC (red, self renewal depicted by red arrow). The CSC by virtue of its infinite proliferative potential will continue to divide as above (dotted arrow). The differentiated cell may undergo a few divisions before terminal differentiation or senescence or cell death as the case may be. The CSC may undergo clonal evolution and acquire even greater tumorigenicity and/or chemoresistance and/or radioresistance (brown). Alternatively, a differentiated cell may de-differentiate and acquire tumorigenicity (black arrow) and be converted to a CSC. (**B**) Clonal Evolution model. Normal cells (blue) acquire mutations stochastically (yellow), and over successive divisions and due to accrual of several mutations these cells become transformed (red) and/or acquire chemo-and/or radio-resistance and can divide indefinitely (dotted arrow).

Head and neck cancer (HNC) is the sixth most common malignancy worldwide [1]. The head and neck is an anatomically complex region that gives rise to a wide variety of cancers varying in phenotype, histology, differentiation, tumorigenicity, and invasiveness [2]. Cancer heterogeneity has been explained by both clonal evolution and cancer stem cell models (Figure 1). In the clonal evolution model, all tumor cells are tumorigenic and heterogeneity arises from genetic or epigenetic changes occurring stochastically within individual clones [3]. The cancer stem cell (CSC) model proposes a hierarchical organization whereby tumor growth is dependent on CSCs that have the capacity for self renewal and to give rise to more differentiated tumor cells, analogous to the role of stem cells in normal tissue [3]. In this setting, small numbers of CSCs can produce a tumor recapitulating the heterogeneity of the original tumor when transplanted into immunocompromised mice [3]. In this model, differentiated daughter cells should not be tumorigenic and cannot recapitulate original tumor heterogeneity. It should be noted that the CSC model does not exclude the possibility that CSCs undergo clonal evolution, and thus these models are not mutually exclusive [3]. Furthermore, it is possible that different tumors follow either the clonal evolution or CSC hypothesis or some combination thereof.

Another important feature of CSCs is resistance to cytotoxic chemotherapy and ionizing radiation [4]. This has profound clinical implications as the clonal evolution model requires treatment directed at all tumor cells, while the CSC model suggests specifically targeting CSCs could affect the entire tumor. In fact, most conventional chemotherapeutic regimens do not kill CSCs and instead enrich the CSC population [4], presumably because CSCs are slowly cycling and over-express efflux pumps capable of extruding these toxic agents [5]. Indeed, the ability to efflux Hoechst 33342 dye has been used to identify CSC populations [6]. Accordingly, it is clinically relevant to understand whether given cancers follow a CSC hierarchy or a clonal evolution hypothesis. For example, Quintana *et al.* recently found that the majority of melanoma cells were tumorgenic, regardless of disease stage [7]. Furthermore, despite using an array of 22 different markers, they were unable to identify a substantial sub-population of non-tumorigenic cells, suggesting that melanoma is a striking example of a non-hierarchically organized cancer [7].

There has been controversy regarding the term ‘cancer stem cell’, as it seems to imply that CSCs have originated through mutation of normal stem cells, however, this is not necessarily true since CSCs might also arise from de-differentiation of tumor cells that acquire alterations imparting “stemness” [8]. Moreover, because ‘CSC’ terminology might imply pluripotency (like embryonic stem cells), some authors prefer the term ‘tumor initiating cells.’ However Gupta *et al.* have argued that since the identification and characterization of CSCs does not rely on their origin and CSCs are oligo-potent, if not pluripotent, these arguments do not contradict a CSC model [8].

A CSC model was first suggested in Acute Myeloid Leukemia (AML), where only a rare population of CD34+ CD38- cells in the peripheral blood collected from AML patients could engraft in immunodeficient mice and recapitulate morphological features of the original malignancy [9]. This landmark discovery, involving isolation of a tumor initiating population by flow cytometry using cell surface markers, paved the way for subsequent experiments in solid tumors, which pose additional challenges requiring dissociation of cells from the tumor. Subsequently, Al-Hajj *et al.* identified the tumor initiating (TI) population from human breast tumors and showed that as few as 100 CD44(+)CD24(-/low)Lineage(-) cells could form tumors while 10,000 cells of the opposite phenotype could not [10]. Work in other solid cancers has since provided evidence of CSCs in ovary, colon, prostate, pancreas and brain tumors [11-18].

In addition to cell surface markers, assays using the ability of CSCs to efflux dyes and aldehyde dehydrogenase 1 (ALDH) activity have been used to identify these cells [4,19]. Efflux pumps of the ATP-binding cassette transporter (ABC transporter) superfamily are overexpressed in CSCs, a property that also enables their isolation by flow cytometry [4]. ALDH is an enzyme responsible for detoxification of intracellular aldehydes [19]. Similar to the high ALDH activity possessed by neural and hematopoietic stem cells, CSCs in AML were found to share this phenotype [19].

Several *in vitro* assays have been used to assess CSC potential, including colony formation in soft agar and sphere formation in serum free media supplemented with growth factors [20]. The sphere formation assay is based on the neurosphere formation assay in which neural stem cells form clonally derived spheres under appropriate culture conditions [20]. The long-term proliferation potential of CSCs is assessed by dissociating tumor spheres into single cells, plating to limiting dilution, then serial passaging [21]. In HNC, CD44+ cells from the Gun-1 cell line express stem cell markers such as CD44 and ABCG2, show increased resistance to chemotherapeutic drugs, and can form spheres *in vitro*, however *in vivo* tumorigenicity of these cells has not been demonstrated [22]. CD133, a marker for both normal and CSC, has also been used in HNC to characterize a tumor initiating cell (TIC) population [21,23-26]. CD133+ cells from the oral SCC cell line UPCI: SCC-016 were characterized to possess cancer stem-like properties of increased tumorigenicity, chemoresistance to paclitaxel, and greater sphere-forming ability than the CD133- population. Moreover these spheres express the stem cell genes Oct-4, and hTERT [27]. The side populations (SP, capable of extruding Hoechst dye) of two highly metastatic HNSCC cell lines M3a2 and M4e had greater sphere formation ability than the non-SP cells and these spheres could be serially passaged [28].

One must bear in mind, however, that while *in vitro* assays can identify populations with stem cell characteristics they cannot replace the most stringent CSC test, the limiting dilution transplantation assay (LDA) in immunocompromised mice that determines the minimum number of CSCs for tumor formation. In fact, the heterogeneity in the fraction of CSCs in a given tumor is likely dependent on cancer-specific characteristics (stage, grade, tissue of origin), contribution of surrounding stromal elements (e.g. fibroblasts) and the immune-competence of the murine host used to assess tumorigenicity [8]. Consistent with previous observations in other cancers, the frequency of tumor initiating cells from HNC increases when transplanted into NOD/SCID mice lacking the interleukin 2 receptor γ chain (IL2Rγ^null^) compared to NOD/SCID mice [3,29]. NOD/SCID mice lacking the interleukin 2 receptor γ chain (IL2Rγ^null^), do not have natural killer cells and are more immunocompromised than NOD/SCID mice, which are only devoid of T and B cells [30].

The study of CSC in HNC has started fairly recently and has been reviewed previously, we have presented here an update to highlight some of the exciting new developments in the area of chemoresistance and metastatic ability of HN-TIC [31-33].

## 2. HNCSC Tumorigenicity

Prince and co-workers published the first report with evidence of CSCs in HNC when they identified a CD44+ population from primary human head and neck cancers that was tumorigenic in nude mice, could be serially passaged, and recapitulated the heterogeneity of the original human tumor [34]. These CD44+ tumor cells expressed basal cell markers, cytokeratin 5 and 14, and the stem cell gene BMI1, consistent with a progenitor function. In contrast, not only could CD44-cells not form tumors in nude mice, but these cells also exhibited features of more differentiated squamous epithelium such as involucrin expression. The fraction of CD44+ cells in the tumor was as high as 10%, and ∼5000 cells was required for tumor formation, suggesting that CD44+ cells are not a pure HNC stem cell population. There are several contradictory reports on the utility of CD44 as a marker for HN-TIC. CD44 is a transmembrane glycoprotein, with several isoforms that differ in their extracellular domains, and serve as a receptor for hyaluronan [35]. Since CD44 variants are known to play a causal role in metastasis in other cancers, therefore the expression of CD44 variants CD44v3, CD44v6 and CD44v10 has been studied in primary tumor and lymph node samples from HNC patients [36,37]. CD44v10 expression correlated with distant metastasis, whereas v3 and v6 were associated with regional and peri-neural metastasis, respectively [37]. Significant association of CD44v6 and v10 with shorter disease-free survival further highlights their importance in HNC [37]. However, other groups have shown that CD44 is expressed by a majority of tumor cells in HNSCC and also by normal HN epithelium as well as benign lesions of the head and neck [38]. A possible explanation for such contradictory results could be the specificity or lack thereof of the CD44 antibodies.

These observations highlight the need for more specific markers or combinations of markers and activity assays for identifying HNCSCs. In breast cancer, for example, the use of a combination of cell surface markers and activity assays, namely CD44+CD24-Lin-ALDH+, has enabled the enrichment of the CSC population such that as few as 20 CSCs form tumors in NOD/SCID mice [39]. Using a similar approach, 1000 CD44+CD24-ALDH+ cells from tumors of oral cancer patients were found to form tumors in SCID mice [40]. In this study, the lowest number of tumor cells implanted was 1000, however, in a separate study as few as 500 ALDH high cells from human HNSCC tumors exhibited tumorigenicity in NOD/SCID mice [41]. It should be noted that in the former study, the lowest number of tumor cells implanted was 1000 so it is not possible to directly compare the two studies in terms of tumorigenicity of the TIC populations.

The combined use of the ALDH and CD44 has recently enabled isolation of HNCSC from primary human tumors wherein 1000 (ALDH+CD44+Lin-) cells were shown to be tumorigenic in SCID mice [42]. These xenografted tumors could be serially passaged in mice, while a 10-fold higher number of ALDH-CD44-Lin-cells could not. Interestingly, these HNCSC were located in close proximity (within 100 μm, the radius of diffusion of oxygen and nutrients from blood vessels) to blood vessels in human tumors, reminiscent of neural and brain tumor stem cells that reside in perivascular niches [42-44]. Analogous to normal stem cells which reside in a protective microenvironment, termed ‘niche’, consisting of a number of cell types such as endothelial cells, and fibroblasts that ensure survival and quiescence, CSCs are also proposed to be dependent on their microenvironment [45,46]. Preliminary experiments suggest that endothelial cells in the niche promote the survival and self renewal of these HNCSC, presumably through secreted growth factors and signaling molecules [42]. These findings, if validated through longer-term *in vivo* experiments to assess the effect of anti-angiogenic therapeutics, may lead to better therapies for HNC patients.

Recently, glucose regulated protein 78 (GRP78) was used to identify HN-TIC from the HNSCC cell line, SAS [47]. GRP78 is an endoplasmic reticulum chaperone protein that is also expressed on the plasma membrane and is essential for survival of embryonic stem cells, presumably by acting in the ER stress response pathway [48]. GRP78 is overexpressed in several cancers including HNSCC, and co-expression of the stem cell marker Nanog with GRP78 is associated with reduced survival of HNSCC patients [47,49,50]. GRP78 is required for tumorigenicty, invasion, and metastasis of HNSCC and as few as 100 plasma membrane GRP78mem+ cells from a HNSCC cell line can form tumors in nude mice [47,51]. While the tumorigenicity of GRP78mem+ from primary human HNSCC remains to be demonstrated, GRP78mem+ cells from SAS, a HNSCC cell line, have stem-cell properties of self-renewal, differentiation and radioresistance. Importantly, knockdown of GRP78 reduces self-renewal and tumorigenicity in nude mice suggesting GRP78 is not merely a marker for HN-TIC but seems to be also involved in their stemness [47]. The molecular mechanism by which GRP78 mediates stemness of HNCSC is an exciting question that needs to be addressed.

## 3. CSC Chemoresistance and Radioresistance

The resistance of CSCs to chemotherapeutic agents has driven much research and CSCs typically overexpress ABC transporters that efflux these agents and are also associated with increased DNA repair and relative quiescence [4]. ABC transporter expression has facilitated CSC isolation by flow cytometry on the basis of their ability to efflux fluorescent dyes like Hoechst [52]. This population of Hoechst dye effluxing cells are referred to as ‘side population’ (SP) cells. SP cells isolated from laryngeal cancer cell lines are radioresistant, have increased *in vivo* tumorigenicity, and can give rise to both SP and non-SP progeny [53]. However, not all SP cells were tumorigenic, and some non-SP cells gave rise to SP cells, suggesting that the ability to efflux Hoechst dye, while characteristic of CSCs, is not limited exclusively to this cell population. This underscores the need to use a combination of cell surface markers in conjunction with dye exclusion or other activity assays to select and define CSC populations. SP cells isolated from aerodigestive squamous cell carcinoma cell lines are more tumorigenic than non-SP cells when transplanted into NOD/SCID mice and overexpress ABC transporters, ABCG2 and ABCC1 [54]. Importantly, the SP population is resistant to the cytotoxic drug mitoxantrone and this resistance can be reversed by chemical inhibition of ABC transporters [54]. SP populations have been characterized from several oral cancer cell lines and primary tumors from patients and were found to be more tumorigenic in mice, express the stem cell gene Bmi1, and ABC transporters in comparison to non-SP cells from the same source [54]. In accordance with the CSC model, the SP cells from an oral cancer cell line could give rise to both SP and non-SP cells under *in vivo* and *in vitro* conditions, whereas the non-SP cells could not generate SP cells [55]. In sum, these observations suggest that inhibition of ABC transporters may be a viable strategy for CSC targeting, and ABCB1 transporter inhibitors are in clinical trials for breast cancer, multiple myeloma and leukemia [56-58].

Because radiation therapy is commonly used to treat HNC, the radioresistance of HNCSCs is of immense interest. Mechanisms of radioresistance have been investigated in brain and breast tumors. Irradiation of tumor bearing mice enriches the rare population of CD133+ glioma stem cells in the brain [59]. Radiation preferentially activates the DNA damage checkpoint and DNA repair pathways in CD133+ cells isolated from human glioblastoma samples compared to CD133- cells, and CD133+ cell radioresistance requires the checkpoint kinases Chk1 and Chk2 [59]. Another mechanism of CSC radioresistance is increased free radical scavenging and reduced levels of reactive oxygen species (ROS); accordingly, pharmacological depletion of ROS scavengers radiosensitizes breast CSCs [60]. Whether HNCSC radioresistance is mediated through similar molecular mechanisms is unknown but holds promise for novel therapeutic strategies for HNC.

## 4. CSCs in Metastasis

Mortality in HNC often results from metastasis to regional lymph nodes and distant organs and CSCs may be critical purveyors of metastasis [61]. Since CSCs have been isolated from primary tumors as well as metastatic sites, an important question is if there are distinct subsets of CSCs that are either stationary or migratory [62]. If these are two distinct pools it will be necessary to therapeutically target both. In a pancreatic cancer cell line, two distinct populations, namely CD133+CXCR4+ and CD133+CXCR4-, are both tumorigenic but only the former migratory cells cause metastasis [14]. Significant progress has been made in colorectal cancer on this subject; screening of several cell surface markers led to the identification of CD26 as a marker for metastatic CSC. The cell surface glycoprotein CD26, is a multifunctional molecule with dipeptidyl peptidase activity and also interacts with extracellular matrix proteins, it plays an important role in tumor progression [63]. Transplantation of only CD26+ cells in the cecal wall of SCID mice gave rise to both cecal tumors and liver metastasis whereas CD26- cells could only form primary tumors and no metastasis occurred [64]. CD26 appears to be directly involved in metastases and CD26 knockdown decreases the expression of proteins related to epithelial to mesenchymal transition (EMT) and *in vitro* invasiveness [64]. This work has profound clinical implications for patient prognosis because the presence of CD26+ cells in primary tumors of patients predicted liver metastasis [64]. Preliminary work in HNSCC cell lines shows the highly metastatic M3a2 and M4e lines have almost 20-times more SP cells compared to their weakly metastatic parental cell line 686LN [28]. However additional work is required to assess the *in vivo* metastatic ability of these populations and answer the questions mentioned above. These SP populations did show stem-like characteristics of chemoresistance, increased *in vitro* invasiveness, and activation of Wnt/β-catenin signaling that is involved in stem cell self-renewal, as compared to non-SP cells [28].

EMT is a developmental program that, when activated in cancer cells, increases invasiveness and motility [65]. Given the importance of EMT in facilitating dissemination of cancer cells, a pertinent question is the molecular mechanism by which these cells in distant tissues self-renew in order to give rise to micrometastasis, and the contribution of EMT to the process of self renewal of metastatic CSCs. The answer to this important question came from the work of Mani and co-workers, who showed that induction of the EMT program in immortalized mammary epithelial cells causes them to acquire stem-like properties in addition to the mesenchymal phenotype [66]. Subsequent work cemented this connection by showing the EMT promoter ZEB1 also promotes stemness by repressing miRNAs that target stemness factors [67].

The connection between EMT and self-renewal has also been explored in HNC. ALDH+ cells isolated from tumors of head and neck patients exhibit stem-like properties such as greater tumorigenicity, sphere formation, and increased radioresistance compared to ALDH- cells [40]. These ALDH+ cells also have features consistent with EMT, namely loss of expression of epithelial markers like E-cadherin and acquisition of mesenchymal markers such as Vimentin and Snail [40]. Snail is a master regulator of EMT and controls invasiveness and metastasis in many cancers [68,69]. The stem-like properties of these ALDH+ HNCs was dependent on Snail expression, suggesting a causal link between EMT and stemness. [40].

Bmi1 is a transcriptional repressor regulating gene expression by changing chromatin structure; it regulates self-renewal of neural, hematopoietic and intestinal stem cells [70-73]. Bmi1 is also involved in HNSC carcinogenesis [55,74]. The ALDH+ HNSCC cells, while exhibiting stemness, also overexpress Bmi1 and Snail in comparison to the ALDH-cells [75]. The tumorigenicity, radioresistance and lung metastasis of these HNSCC ALDH+ cells was dependent on Bmi1 [75]. It is pertinent to point out that this is the first report of lung metastasis of HNC stem-like cells [75]. Clinically, co-expression of ALDH, Bmi1 and Snail predicted the worst prognosis in HNC patients [75]. This study further strengthened the link between EMT and stemness in HNC.

Seminal work by Yang and co-workers has provided the mechanistic link between EMT and self renewal in HNSCC [76]. The stemness factor Bmi1 is directly regulated by the EMT regulator, Twist. Using HNSCC cell lines they show that the transcription factors Bmi1 and Twist act cooperatively to regulate p16INK4a and E-cadherin, mediating self renewal and EMT, respectively. These observations provide the vital explanation underlying the worse prognosis of HNSCC patients overexpressing both Bmi1 and Twist as compared to those expressing either protein alone.

## 5. Conclusion and Future Perspectives

Research on HNCSC started fairly recently in 2007, with the use of CD44 as a marker for isolation of stem cells from human HN tumors [34]. Subsequent work has expanded the repertoire of cell surface markers and activity assays used for HNCSC isolation to include CD133, ALDH, Hoechst dye exclusion (SP), and GRP78 [27,40,47,54]. Proteomic analysis of the SAS HNSCC cell line grown in regular media and sphere cells (presumably a TIC population) led to identification of GRP78 as a putative HNCSC marker [47]. This study highlights the use of proteomics to identify novel markers. Additional markers are needed to isolate pure populations of HNCSC and combinations of makers and activity assays may provide further enrichment of the CSC pool.

Molecular characterization of gene expression by microarray analysis of the HNCSC populations has provided evidence of activation of the EMT program and of Wnt/β-catenin signaling in these cells [28,40]. The Wnt/β-catenin signaling pathway is involved in diverse aspects of normal stem cell biology such as maintenance of pluripotency, differentiation and proliferation [77-79]. This signaling pathway has also been implicated in CSCs, and inhibitors of this pathway are in clinical trials in several cancers [80]. These observations need to be extended to HNCSCs isolated from primary human tumor samples to identify additional signaling pathways that are preferentially activated in HNCSC, and may constitute novel therapeutic targets. In summary, HNC seems to follow the CSC model of tumorigenesis, though additional work is necessary for refinement of molecular mechanisms. Observations made in HNSCC cell lines need to be confirmed in patient samples.
